# Therapeutic Potential of Multilineage-Differentiating Stress-Enduring Cells for Osteochondral Repair in a Rat Model

**DOI:** 10.1155/2017/8154569

**Published:** 2017-10-29

**Authors:** Elhussein Elbadry Mahmoud, Naosuke Kamei, Ryo Shimizu, Shohei Wakao, Mari Dezawa, Nobuo Adachi, Mitsuo Ochi

**Affiliations:** ^1^Department of Orthopaedic Surgery, Integrated Health Sciences, Institute of Biomedical and Health Sciences, Hiroshima University, Hiroshima, Japan; ^2^Department of Surgery, Faculty of Veterinary Medicine, South Valley University, Qena 83523, Egypt; ^3^Medical Center for Translational and Clinical Research, Hiroshima University Hospital, Hiroshima, Japan; ^4^Department of Stem Cell Biology and Histology, Graduate School of Medicine, Tohoku University, Sendai, Japan

## Abstract

Multilineage-differentiating stress-enduring (Muse) cells are stage-specific embryonic antigen-3 (SSEA-3) positive cells existing in mesenchymal stem cell (MSC) populations. Muse cells have the pluripotency to differentiate into all germ layers as embryonic stem cells. In this study, we aimed to investigate the efficacy of Muse cell transplantation for osteochondral defect repair. Muse cells were isolated from human bone marrow MSCs. An osteochondral defect was created in the patellar groove of immunodeficient rats. After this, cell injection was performed, whereby rats were divided into 3 groups: the control group, the rats of which were given a PBS injection; the non-Muse group, which comprised 5 × 10^4^ SSEA-3 negative non-Muse cells; and the Muse group, which comprised 5 × 10^4^ SSEA-3 positive Muse cells. The white repaired tissue had a mostly smooth homogenous surface at 12 weeks after treatment in the Muse group, while no repair tissue was detected in the control and non-Muse groups. Histological assessments showed better repair at the cartilage defect sites in the Muse group compared to the other groups at 4 and 12 weeks after treatment. Muse cells could be a new promising cell source for the treatment of osteochondral defects.

## 1. Introduction

Cartilage lesions cause joint disability, due to both their limited intrinsic capacity to repair themselves and the repercussion of reduced joint function, which equates to significant disability especially among elderly patients [1].

Several clinical trials using methods such as marrow-stimulating techniques and osteochondral graft have been conducted in an attempt to improve cartilage repair, but success has been limited. In 1994, Brittberg et al. performed the first generation of cell therapy named autologous chondrocyte implantation (ACI) [2], and Ochi et al. modified ACI, using atelocollagen gel in combination with chondrocytes to produce a good clinical outcome [3]. However, success is still limited, due to the morbidity of the intact cartilage, dedifferentiation, and the two-stage surgical procedure.

Over the past decade, mesenchymal stem cells (MSCs) have been widely used as a cell-based therapy for clinical application; thanks to the fact that they can be easily isolated, they are very accessible from different tissues, and they have a high rate of expansion and proliferation. Furthermore, triploblastic differentiation can be widely performed [4]. Among heterogeneous crude populations of MSCs, there are novel pluripotent stem cells, which are initially isolated from human bone marrow and dermal fibroblasts under cellular stress conditions (low nutrition or trypsin incubation) and are called multilineage-differentiating stress-enduring (Muse) cells. Muse cells have the pluripotency to differentiate into all germ layers as embryonic stem cells. These are double positive expressed to CD105 and stage-specific embryonic antigen-3 (SSEA-3). Cells negative to the SSEA-3 marker in the MSC population are called non-Muse cells [5]. Recently, Muse cells were isolated from human adipose tissue by another research group [6] and also were isolated from commercially available human adipose stem cells (ASCs) [7]. Muse cells were not only isolated from humans but also reported in a goat model [8]. The development of cell transplantation has seen recent studies use adipose- and bone marrow-Muse cells to treat skin ulcers and brain infarction, respectively [9, 10]. In this study, we aimed to clarify the therapeutic potential of human Muse cells compared with non-Muse cells for the repair of osteochondral defects in the immunodeficient rat model.

## 2. Materials and Methods

All procedures of this study were performed according to the guide for animal experimentation, Hiroshima University. All protocols were approved and performed by the Committee of Research Facilities for Laboratory Animal Sciences, Graduate School of Biomedical Sciences, Hiroshima University.

### 2.1. Cell Source

After purchasing human bone marrow MSCs (hBMSCs; Lonza, Basel, Switzerland), they were cultured at 37°C, 5% CO_2_ in minimal essential medium eagle (*α*-MEM) containing 10% fetal bovine serum (FBS), 0.1 mg/ml kanamycin, and 1% Glutamax (Thermo Fisher Scientific, Waltham, MA). The hBMSCs were subcultured at a ratio of 1 : 2 after reaching 90–100% confluence using 0.25% trypsin-ethylenediaminetetraacetic acid. According to the previous protocol designed by Kuroda et al. [11], briefly, hBMSCs were separated into Muse cells (SSEA-3+) and non-Muse cells (SSEA-3−), according to whether or not there was expression of SSEA-3. hBMSCs were incubated with SSEA-3 antibody (1 : 100; Merck Millipore, Darmstadt, Germany), detected by allophycocyanin-conjugated antirat IgM (Jackson ImmunoResearch, West Grove, PA) in the antibody diluents and sorted by Special Order Research Products FACSAria II (Becton Dickinson, Franklin Lakes, NJ).

### 2.2. Cell Injection into the Defect Site

This study was performed on 16 immunodeficient rats (32 knees) (F344/NJcl-rnu/rnu) aged 10 weeks. An osteochondral defect (2 mm diameter, 2 mm depth) was created bilaterally in the patellar groove of the femur using a commercially available metallic drill which had a globe-shaped tip of 1 mm diameter. Immediately after closure of the knee joint, the rats' knees were unequally distributed into 3 groups (Table 1): control group—PBS injection; non-Muse group—an intra-articular injection of non-Muse cells (5 × 10^4^); and Muse group—an intra-articular injection of Muse cells (5 × 10^4^). Cells were suspended in 50 *μ*l of PBS.

### 2.3. Macroscopic and Histological Assessment

At 4 and 12 weeks after treatment, rats were sacrificed by means of an intraperitoneal injection of a lethal dose of pentobarbital sodium, then femoral condyles were evaluated macroscopically using macroscopic scoring system with 14 as the best and 0 as the worst (Table 2), produced by Wayne et al. [12, 13]. Then, repaired tissue was fixed in Paraformaldehyde phosphate buffered solution 4% for 1 day, and samples were decalcified with EDTA 10% (Nacalai Tesque Inc., Kyoto, Japan) for 4 weeks, after which they were embedded in paraffin blocks. The samples were cut into 5 *μ*m sections sagittally. For histological evaluation, sections were stained with safranin O/fast green stain (Muto Pure Chemicals Co. Ltd., Japan) to produce histological scoring on the Sellers scale (Table 3) [14]. The hematoxylin and eosin (H&E) staining was also used to assess the cell density of repair tissue.

### 2.4. Immunostaining

At 4 and 12 weeks after treatment, the sections were pretreated with antigen retrieval reagent (Immunoactive, Matsunami Glass Ind., Osaka, Japan) and immersed in 0.3% H_2_O_2_ to block endogenous peroxidase activity. The sections were blocked with blocking solution (Protein Block Serum-Free; Dako, Carpinteria, CA) and incubated with mouse monoclonal antibodies directed against type I collagen (1 : 250, Daiichi Fine Chemical, Toyama, Japan) and type II collagen (1 : 250, Daiichi Fine Chemical). The reaction for visualization was performed using an avidin-biotin peroxidase system (Vectastain Elite ABC kit; Vector Laboratories, Burlingame, CA), and the sections were colored with 3,3′-diaminobenzidine (Peroxidase Substrate Kit, Vector Laboratories Inc.).

### 2.5. Statistical Analysis

Histological scoring was analyzed by the Kruskal-Wallis and Steel-Dwass tests, with a 95% confidence interval. Values of *P* < 0.05 were considered significant.

## 3. Results

### 3.1. Macroscopic Findings

**Figure 1:**
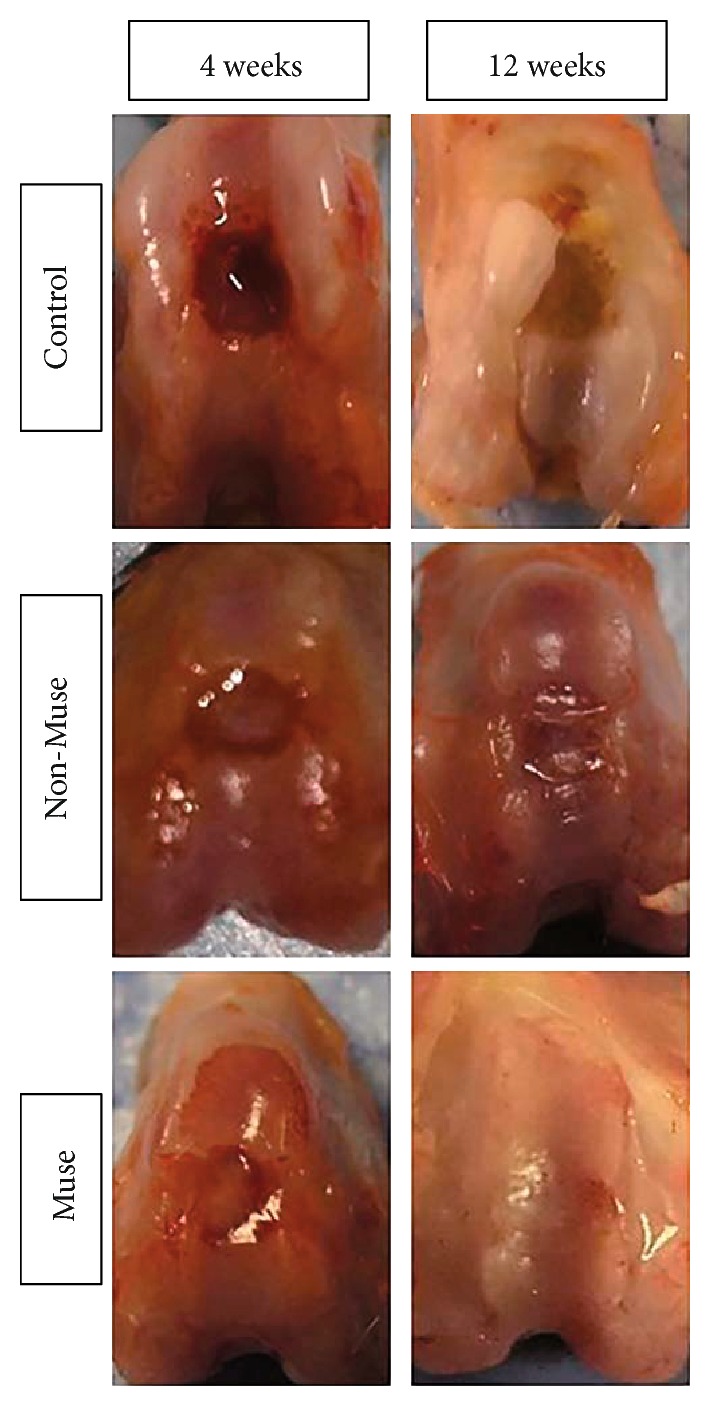
Macroscopic findings of the repaired tissue in the control, non-Muse, or Muse groups at 4 and 12 weeks, with complete filling of the defect with white tissue at the same level as normal tissue in the Muse groups at 12 weeks.

**Figure 2:**
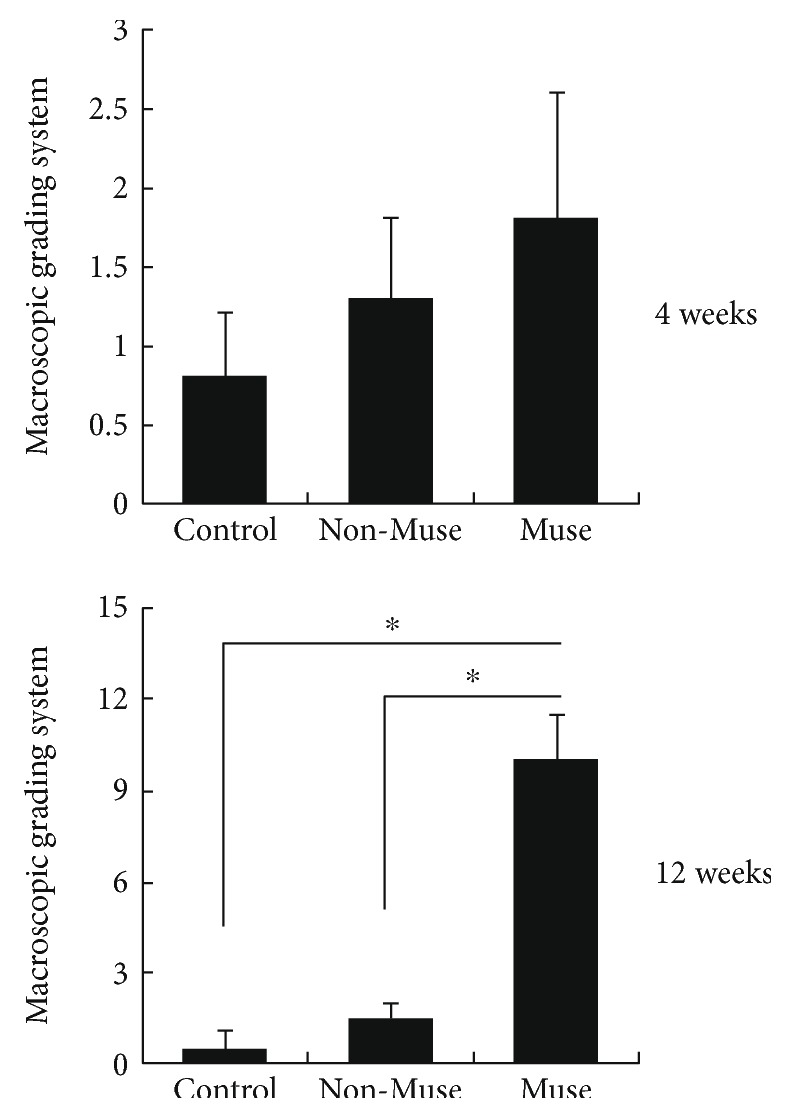
The macroscopic grading system revealed no significant difference between the groups at 4 weeks. However, at 12 weeks, the Muse group showed significantly better results than the other groups. ^∗^*P* < 0.05.

Repair tissue was not detected, and defect margins were easily identified in the patellar groove of the control and non-Muse groups. Moreover, at 4 and 12 weeks, osteoarthritic changes including degeneration of the adjacent cartilage increased in the control group compared with both Muse and non-Muse cells of the experimental groups. At 12 weeks, the depth of the defect was reduced in the non-Muse group which was filled with brown tissue, while in the Muse group, there was evidence of complete filling of the defect with white tissue, which appeared to have a smooth homogeneous surface in accordance with the surrounding tissue, making it hard to clearly identify the defect margins (Figure 1). On macroscopic scoring, there was no significant difference among the three groups at 4 weeks after treatment (control 0.8 ± 0.4, non-Muse 1.3 ± 0.5, and Muse 1.8 ± 0.8). However, the macroscopic result of the Muse group was significantly better than that of the other groups at 12 weeks after treatment, determined by the defect filling (control 0.5 ± 0.6, non-Muse 1.5 ± 0.5, and Muse 10.0 ± 1.5) (Figure 2). Macroscopic scores of the individual parameters were shown in Table 4.

### 3.2. Histological Findings and Scoring

**Figure 3:**
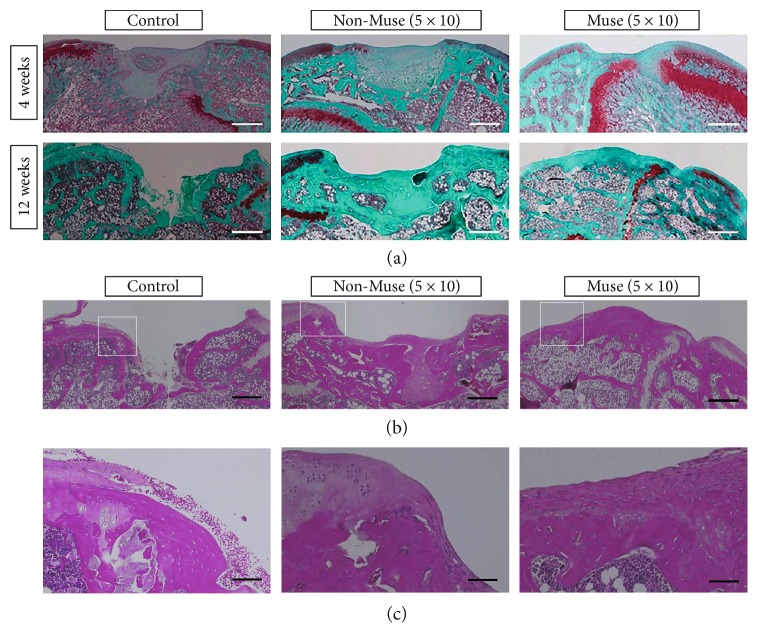
(a) At 4 and 12 weeks, histological evaluation of the repaired tissue using safranin O/fast green stain. (b, c) H&E staining at 12 weeks. Scale bars: (a, b) 500 *μ*m, (c) 100 *μ*m.

**Figure 4:**
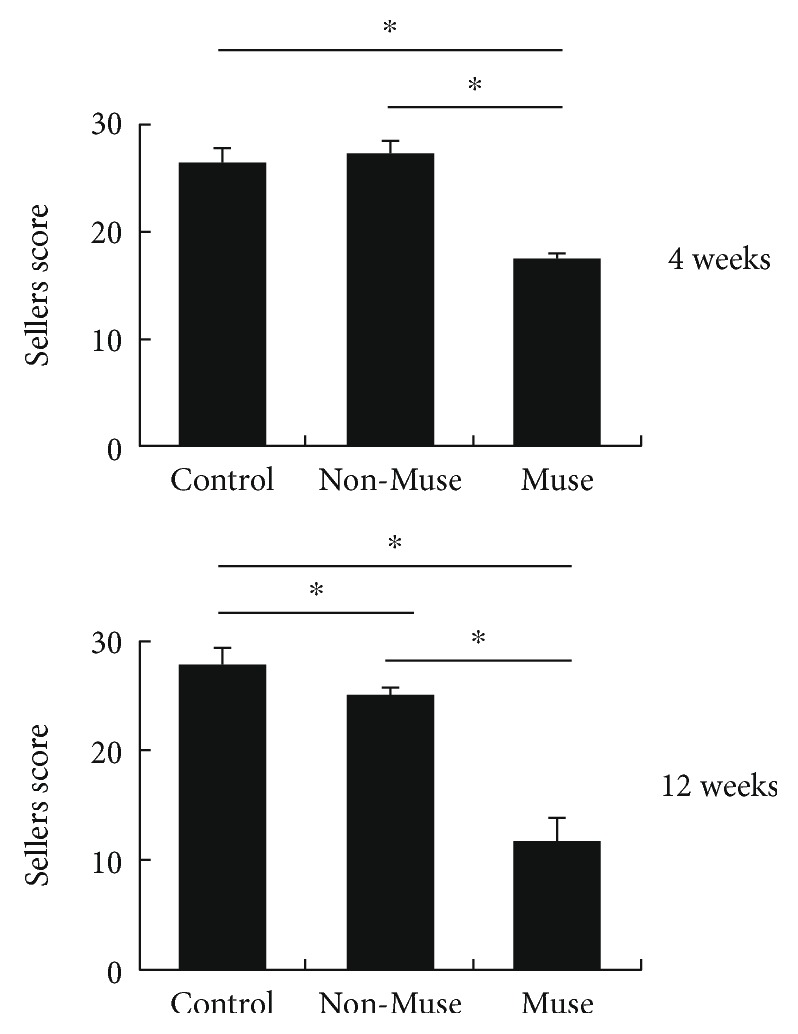
Histological scoring using Sellers scale at 4 and 12 weeks, with histologically significant difference in the Muse groups, unlike in either the non-Muse or control groups. ^∗^*P* < 0.05.

At time of sacrifice in both the control and non-Muse groups, there was a small amount of fibrous tissue but no repair tissue in the defect site. In the Muse group at 4 weeks, there was partial repair of the defect involving repair of the subchondral bone without replacement of cartilage. However, at 12 weeks, repair of the osteochondral defect was confirmed with complete repair of the subchondral bone, but it was covered with fibrous tissue, and in addition to integration, an osteochondral junction was observed (Figure 3(a)). At 4 and 12 weeks, the following results were recorded based on the Sellers scale: control (4 W 26.2 ± 1.6, 12 W 27.8 ± 1.5), non-Muse (4 W 27.2 ± 1.2, 12 W 25 ± 0.6), and Muse groups (4 W 17.4 ± 0.6, 12 W 11.8 ± 2.0). The non-Muse group produced a significantly better result than the control group at 12 weeks after treatment. Additionally, the score in the Muse group was significantly better than that in the other groups both at 4 and 12 weeks after treatment (Figure 4). Sellers scores of the individual parameters were shown in Table 5. H&E staining at 12 weeks showed higher cell density of repair tissue in the Muse group compared with the other groups (Figures 3(b) and 3(c)).

### 3.3. Immunostaining for Collagen Type I and II

**Figure 5:**
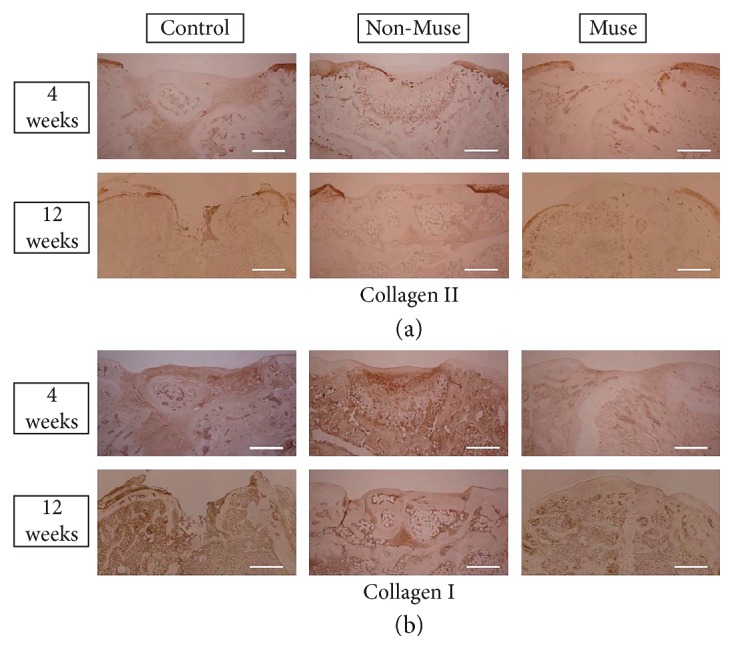
Immunohistochemical staining of collagen type I and II. There was no detection of collagen type I and II in the repaired tissue of the Muse group. Scale bars 500 *μ*m.

At 4 and 12 weeks after treatment, the surface of the injured area was not stained with collagen type II in any of the groups. In contrast, the surface of the injured area was stained with collagen type I in all groups except for the Muse group at 12 weeks. In the Muse group at 12 weeks, the injured area was covered with collagen type I and type II negative tissue (Figure 5).

## 4. Discussion

In the present study, we demonstrated that an intra-articular injection of Muse cells derived from hBMSCs improved the repair of an osteochondral defect compared with that of non-Muse MSCs.

To date, there has been no in vivo study to evaluate the effect of Muse cells as a new generation of cell therapy especially for the mesodermal lineage like chondrocytes, osteocytes, and adipocytes regarding musculoskeletal disorders. Recent studies have shown that Muse cells derived from a population of ASCs suspended in hyaluronic acid have a beneficial therapeutic effect on the healing of skin ulcers with diabetes under stressful cellular conditions [9]. Also, bone marrow-Muse cells have been shown to integrate into an infarcted mouse brain and to differentiate into Tuj-1- and NeuN-expressing cells for the replacement of lost neurons, while non-Muse cells produce trophic factors which are not detected in the brain tissue [10]. In the case of the endodermal lineage, Katagiri et al. detected that bone marrow-Muse cells are able to repair liver components after an intravenous injection, with the capacity to integrate an injured area after partial hepatectomy [15]. The present study is the first report to show the efficacy of Muse cells on the osteochondral repair.

Previous studies have revealed that MSC populations have pleiotropic actions, which enable integration into damaged tissues and differentiation into specific cells although the mechanism of tissue homing is not known [16]. Previous reports which coincide with our hypothesis revealed identical parameters of macroscopic findings, including defect filling and color which correlated to the quality of the repaired tissue [17]. In the present study, good repair of the defect was confirmed macroscopically in the Muse group, although our hypothesis is contradicted by the fact that cartilage repair was not satisfactory histologically. However, despite cartilage repair in our study not being as perfect as subchondral repair (Sellers scores of subchondral bone repair; 4.0 ± 0 in control group, 3.0 ± 0 in non-Muse group, and 0.3 ± 0.5 in Muse group); it is worth conducting extensive further research into the chondrogenic potential of Muse cells, especially considering their unique properties such as pluripotency and the lack of teratoma formation, in contrast to iPS and ES cells. Wakitani et al. proved that it is impossible to repair the cartilage of a knee joint which has been destroyed by ES cells due to tumor formation [18]. Muse cells display low proliferative and telomerase activities, as well as lower expression of what is called Yamanaka factors compared with iPS cells, which was slightly more pronounced than in non-Muse cells [5]. These results revealed an intermediate balance of Muse cells for pluripotency and teratogenesis with the elimination of teratoma information.

From the aforementioned results, we speculate that bone marrow-Muse cells have tended to demonstrate osteogenic rather than chondrogenic potential. Similarly, bone marrow-MSCs have a greater affinity to osteogenic than to chondrogenic potential. Unlike non-Muse cells, adipose-Muse cells are highly efficient either for adipocyte, hepatocyte, or neuronal induction. In addition, unlike BM- and dermal-Muse cells, adipose-Muse cells exhibit a higher mesodermal lineage, whereby they express osteogenic, myogenic, and adipogenic genes [7]. However, adipose-Muse cells have a relatively low expression of many genes involved in tissue development, cellular function, and cell cycling compared with ASCs. Pluripotency markers in Muse cells derived from fibroblasts and bone marrow were upregulated [5]. Also, gene levels related to the ectodermal lineage were upregulated in the case of BM- and dermal-Muse cells more than in the case of adipose-Muse cells [7]. Differentiation of Muse cells is not very high, and both cytokines and trophic factors when combined with Muse cells lead to more than 90% of Muse cells being differentiated into the targeted cells [16].

The use of non-Muse cells producing trophic factors must be catalytically required, in order for there to be an improvement in the chondrogenesis of Muse cells. A previous study revealed that Muse cells derived from ASCs secrete numerous growth factors such as TGF-*β*, bFGF, and TNF-*α*, especially under hypoxic conditions (1% O_2_) [12]. An intra-articular injection of MSCs is the most convenient method for cartilage repair, although success has not been satisfactory, because the presence of Muse cells has not exceeded 1% of the BM-MSC population spread over the whole joint, with a low survival rate (<3%) in a high-stress environment such as myocardial infarction, ischemia, and experimental stroke [19, 20]. In the future, Muse cells may produce a better clinical outcome than MSCs due to their capacity to integrate within an unfavorable environment, in contrast to MSCs with their propensity to cell death under the same conditions [21]. Therefore, further investigation is necessary to detect the accurate ratio between Muse/non-Muse cells.

Potential limitations have become evident through our study. A small number of animals was used in each group but can be avoided in further experiments. Obtaining MSCs from bone marrow requires an extremely invasive method. Macroscopic and histological evaluations were unthematic because of unblinded assessment. Even so, Muse cell acts as repair cells and we cannot simply achieve good repair to resemble the native one.

## 5. Conclusions

An intra-articular injection of Muse cells is a promising method to repair an osteochondral defect, especially subchondral bone covered by fibrous tissue.

## Figures and Tables

**Table 1:** Distribution of the number of rats' knees per group with 2 different time points 4 and 12 weeks.

Time point	Control group	Non-Muse group	Muse group	Total
4 weeks	5	6	5	16
12 weeks	4	6	6	16
Total	9	12	11	**32**

**Table 2:** Macroscopic scoring system.

Coverage	
>75% fill	4
50–75% fill	3
25–50% fill	2
<25% fill	1
No fill	0
Neocartilage color	
Normal	4
25% yellow/brown	3
50% yellow/brown	2
75% yellow/brown	1
100% yellow/brown	0
Defect margins	
Invisible	4
25% circumference visible	3
50% circumference visible	2
75% circumference visible	1
Entire circumference visible	0
Surface	
Smooth/level with normal	4
Smooth but raised	3
Irregular 25–50%	2
Irregular 50–75%	1
Irregular >75%	0

**Table 3:** Sellers scale for histological assessment of the repaired tissue.

*Filling of the defect relative to surface of normal adjacent cartilage*	
111%–125%	1
91%–110%	0
76%–90%	1
51%–75%	2
26%–50%	3
<25%	4
*Integration of repair tissue with surrounding articular cartilage*	
Normal continuity and integration	0
Decreased cellularity	1
Gap or lack of continuity on one side	2
Gap or lack of continuity on two sides	3
*Matrix staining with safranin O/fast green*	
Normal	0
Slightly reduced	1
Moderately reduced	2
Substantially reduced	3
None	4
*Cellular morphology (choose first between a, b, c, and d)*	
(a) Normal	0
(b) Mostly round cells with the morphology of chondrocytes	
>75% of tissue with columns in radial zone	0
25%–75% of tissue with columns in radial zone	1
<25% of tissue with columns in radial zone (disorganized)	2
(c) 50% round cells with the morphology of chondrocytes	
>75% of tissue with columns in radial zone	2
25%–75% of tissue with columns in radial zone	3
<25% of tissue with columns in radial zone (disorganized)	4
(d) Mostly spindle-shaped (fibroblast-like) cells	5
*Architecture within entire defect (not including margins)*	
Normal	0
1–3 small voids	1
1–3 large voids	2
>3 large voids	3
Clefts or fibrillations	4
*Architecture of surface*	
Normal	0
Slight fibrillation or irregularity	1
Moderate fibrillation or irregularity	2
Severe fibrillation or disruption	3
*Percentage of new subchondral bone*	
90%–100%	0
75%–89%	1
50%–74%	2
25%–49%	3
<25%	4
*Formation of tidemark*	
Complete	0
75%–99%	1
50%–74%	2
25%–49%	3
<25%	4

**(a) Coverage:** 

4 W			12 W		
Control	Non-Muse	Muse	Control	Non-Muse	Muse
0.8 ± 0.4	1.3 ± 0.5	1.8 ± 0.8^∗^	0.5 ± 0.6	1.5 ± 0.5^∗^	3.5 ± 0.5^∗^

**(b) Neocartilage color:** 

4 W			12 W		
Control	Non-Muse	Muse	Control	Non-Muse	Muse
0 ± 0	0 ± 0	0 ± 0	0 ± 0	0 ± 0	2.2 ± 0.8^∗^

**(c) Defect margins:** 

4 W			12 W		
Control	Non-Muse	Muse	Control	Non-Muse	Muse
0 ± 0	0 ± 0	0 ± 0	0 ± 0	0 ± 0	2.3 ± 0.5^∗^

**(d) Surface:** 

4 W			12 W		
Control	Non-Muse	Muse	Control	Non-Muse	Muse
0 ± 0	0 ± 0	0 ± 0	0 ± 0	0 ± 0	2.0 ± 0^∗^

^∗^Significantly better than control (*P* < 0.05).

**(a) Filling of the defect relative to surface of normal adjacent cartilage:** 

4 W			12 W		
Control	Non-Muse	Muse	Control	Non-Muse	Muse
2.0 ± 0.7	2.0 ± 0	1.2 ± 0.4^∗^	2.0 ± 0.8	2.2 ± 0.4	1.0 ± 1.1^∗^

**(b) Integration of repair tissue with surrounding articular cartilage:** 

4 W			12 W		
Control	Non-Muse	Muse	Control	Non-Muse	Muse
3.0 ± 0	3.0 ± 0	3.0 ± 0	3.0 ± 0	3.0 ± 0	0 ± 0^∗^

**(c) Matrix staining with safranin O/fast green:** 

4 W			12 W		
Control	Non-Muse	Muse	Control	Non-Muse	Muse
4.0 ± 0	4.0 ± 0	4.0 ± 0	4.0 ± 0	4.0 ± 0	4.0 ± 0

**(d) Cellular morphology (choose first between a, b, c, and d):** 

4 W			12 W		
Control	Non-Muse	Muse	Control	Non-Muse	Muse
5.0 ± 0	5.0 ± 0	5.0 ± 0	5.0 ± 0	5.0 ± 0	5.0 ± 0

**(e) Architecture within the entire defect (not including margins):** 

4 W			12 W		
Control	Non-Muse	Muse	Control	Non-Muse	Muse
2.8 ± 1.3	3.5 ± 1.2	0.2 ± 0.4^∗^	3.0 ± 1.1	1.0 ± 0^∗^	0 ± 0^∗^

**(f) Architecture of surface:** 

4 W			12 W		
Control	Non-Muse	Muse	Control	Non-Muse	Muse
1.6 ± 0.9	2.0 ± 0.6	1.2 ± 0.4	2.8 ± 0.5	3.0 ± 0	1.0 ± 0^∗^

**(g) Percentage of new subchondral bone:** 

4 W			12 W		
Control	Non-Muse	Muse	Control	Non-Muse	Muse
3.8 ± 0.4	4.0 ± 0	1.0 ± 0.7^∗^	4.0 ± 0	3.0 ± 0^∗^	0.3 ± 0.5^∗^

**(h) Formation of tidemark:** 

4 W			12 W		
Control	Non-Muse	Muse	Control	Non-Muse	Muse
4.0 ± 0	4.0 ± 0	1.8 ± 1.1^∗^	4.0 ± 0	3.8 ± 0.4	0.5 ± 0.5^∗^

^∗^Significantly better than control (*P* < 0.05).

## References

[1] Mobasheri A., Csaki C., Clutterbuck A. L., Rahmanzadeh M., Shakibaei M. (2009). Mesenchymal stem cells in connective tissue engineering and regenerative medicine: applications in cartilage repair and osteoarthritis therapy. *Histology and Histopathology*.

[2] Brittberg M., Lindahl A., Nilsson A., Ohlsson C., Isaksson O., Peterson L. (1994). Treatment of deep cartilage defects in the knee with autologous chondrocyte transplantation. *The New England Journal of Medicine*.

[3] Ochi M., Uchio Y., Kawasaki K., Wakitani S., Iwasa J. (2002). Transplantation of cartilage-like tissue made by tissue engineering in the treatment of cartilage defects of the knee. *The Journal of Bone & Joint Surgery*.

[4] Tamai K., Yamazaki T., Chino T. (2011). PDGFRα-positive cells in bone marrow are mobilized by high mobility group box 1 (HMGB1) to regenerate injured epithelia. *Proceedings of the National Academy of Sciences of the United States of America*.

[5] Kuroda Y., Kitada M., Wakao S. (2010). Unique multipotent cells in adult human mesenchymal cell populations. *Proceedings of the National Academy of Sciences of the United States of America*.

[6] Heneidi S., Simerman A. A., Keller E. (2013). Awakened by cellular stress: isolation and characterization of a novel population of pluripotent stem cells derived from human adipose tissue. *PLoS One*.

[7] Ogura F., Wakao S., Kuroda Y. (2014). Human adipose tissue possesses a unique population of pluripotent stem cells with nontumorigenic and low telomerase activities: potential implications in regenerative medicine. *Stem Cells and Development*.

[8] Yang Z., Liu J., Liu H. (2013). Isolation and characterization of SSEA3^+^ stem cells derived from goat skin fibroblasts. *Cellular Reprogramming*.

[9] Kinoshita K., Kuno S., Ishimine H. (2015). Therapeutic potential of adipose-derived SSEA-3-positive Muse cells for treating diabetic skin ulcers. *Stem Cells Translational Medicine*.

[10] Yamauchi T., Kuroda Y., Morita T. (2015). Therapeutic effects of human multilineage-differentiating stress enduring (MUSE) cell transplantation into infarct brain of mice. *PLoS One*.

[11] Kuroda Y., Wakao S., Kitada M., Murakami T., Nojima M., Dezawa M. (2013). Isolation, culture and evaluation of multilineage-differentiating stress-enduring (Muse) cells. *Nature Protocols*.

[12] Wayne J. S., McDowell C. L., Shields K. J., Tuan R. S. (2005). In vivo response of polylactic acid–alginate scaffolds and bone marrow-derived cells for cartilage tissue engineering. *Tissue Engineering*.

[13] Cui W., Wang Q., Chen G. (2011). Repair of articular cartilage defects with tissue-engineered osteochondral composites in pigs. *Journal of Bioscience and Bioengineering*.

[14] Sellers R. S., Peluso D., Morris E. A. (1997). The effect of recombinant human bone morphogenetic protein-2 (rhBMP-2) on the healing of full-thickness defects of articular cartilage. *The Journal of Bone & Joint Surgery- American Volume*.

[15] Katagiri H., Kushida Y., Nojima M. (2016). A distinct subpopulation of bone marrow mesenchymal stem cells, Muse cells, directly commit to the replacement of liver components. *American Journal of Transplantation*.

[16] Kuroda Y., Kitada M., Wakao S., Dezawa M. (2011). Bone marrow mesenchymal cells: how do they contribute to tissue repair and are they really stem cells?. *Archivum Immunologiae et Therapiae Experimentalis*.

[17] Goebel L., Orth P., Muller A. (2012). Experimental scoring systems for macroscopic articular cartilage repair correlate with the MOCART score assessed by a high-field MRI at 9.4 T – comparative evaluation of five macroscopic scoring systems in a large animal cartilage defect model. *Osteoarthritis and Cartilage*.

[18] Wakitani S., Takaoka K., Hattori T. (2003). Embryonic stem cells injected into the mouse knee joint form teratomas and subsequently destroy the joint. *Rheumatology*.

[19] Oh J. S., Kim K. N., An S. S. (2011). Cotransplantation of mouse neural stem cells (mNSCs) with adipose tissue-derived mesenchymal stem cells improves mNSC survival in a rat spinal cord injury model. *Cell Transplantation*.

[20] Uchida H., Morita T., Niizuma K. (2016). Transplantation of unique subpopulation of fibroblasts, Muse cells, ameliorates experimental stroke possibly via robust neuronal differentiation. *Stem Cells*.

[21] Dezawa M. (2016). Muse cells provide the pluripotency of mesenchymal stem cells: direct contribution of Muse cells to tissue regeneration. *Cell Transplantation*.

